# Sustaining the Elimination of Visceral Leishmaniasis: A Secondary Data Analysis of the National Kala-Azar Elimination Programme in Malda District, West Bengal, India (2018-2022)

**DOI:** 10.7759/cureus.112553

**Published:** 2026-07-12

**Authors:** Tanudipta Halder, Priya Debnath, Jayita Pal, Manoj Ghosh

**Affiliations:** 1 Department of Research Methodology and Biostatistics, L. R. Shah Homoeopathy College, Rajkot, IND; 2 Department of Public Health, M.K. University, Patan, IND; 3 Department of Public Health, Institute of Public Health, Kalyani, IND; 4 Department of Community Medicine, L. R. Shah Homoeopathy College, Rajkot, IND; 5 Department of Epidemiology and Public Health, All India Institute of Hygiene and Public Health, Kolkata, IND

**Keywords:** active case detection, disease surveillance, indoor residual spraying, post kala-azar dermal leishmaniasis, program evaluation, visceral leishmaniasis (vl)

## Abstract

Background

Visceral leishmaniasis (VL), commonly known as *kala-azar*, remains a public health concern in several endemic regions of India. Sustaining elimination requires continuous surveillance and programmatic monitoring. This study assessed the progress of the National Kala-Azar Elimination Programme in Malda district, West Bengal, during 2018-2022.

Methods

A descriptive cross-sectional study based on secondary analysis of routine programme data was conducted using records maintained by the Chief Medical Officer of Health, Malda. Data on VL and post-*kala-azar* dermal leishmaniasis (PKDL) cases, mortality, active case detection, treatment outcomes, vector control activities, and logistics management were extracted and analysed using Microsoft Excel 2019 (Microsoft Corporation, Redmond, WA). Descriptive statistics were used to summarize trends and programme performance indicators.

Results

VL cases declined from 19 in 2018 to seven in 2022, indicating sustained control of disease transmission. In contrast, PKDL cases increased from 40 in 2018 to 55 in 2022 and constituted the majority of reported *kala-azar* cases in 2022. Adults aged >15 years accounted for 89.9% of VL cases and 92.2% of PKDL cases. Males represented the majority of cases in most years, while tribal populations contributed 40-71% of annual reported cases. All 15 endemic blocks maintained the elimination target throughout the study period, with 11 blocks reporting no VL cases in 2022. Treatment completion exceeded 90% annually, indoor residual spraying achieved population coverage above 91%, and active case detection continued to identify confirmed cases among screened populations.

Conclusion

Malda district successfully sustained *kala-azar* elimination status throughout the study period. Continued surveillance, early detection and treatment of PKDL, follow-up of treated VL patients, and targeted interventions in tribal and historically endemic areas are essential to prevent resurgence and sustain elimination gains.

## Introduction

Globally, visceral leishmaniasis (VL), commonly known as *kala-azar* (KA), remains a major neglected tropical disease, with transmission concentrated in a few endemic countries, while post-*kala-azar* dermal leishmaniasis (PKDL) continues to play a pivotal role in sustaining *Leishmania donovani* transmission by serving as a long-term human reservoir [1]. In India, substantial progress towards KA elimination has been achieved through intensified vector control, early diagnosis, and effective treatment; however, PKDL remains a significant challenge due to delayed detection and its contribution to ongoing transmission in endemic foci [1,2]. West Bengal, one of the historically endemic states in eastern India, has experienced a marked decline in KA incidence, yet sporadic KA and PKDL cases continue to be reported, underscoring the need for sustained surveillance, active case detection, and effective PKDL management to consolidate elimination gains and prevent disease resurgence [2].

PKDL is a dermal manifestation that may occur months to years after successful treatment of VL. Patients with PKDL are considered an important reservoir of *Leishmania* parasites and play a critical role in sustaining disease transmission within communities. Consequently, early detection and effective treatment of PKDL are essential components of VL elimination strategies [3,4].

The Government of India launched a centrally sponsored Kala-azar Control Programme in 1990-91 under the National Vector Borne Disease Control Programme (NVBDCP). With the regional commitment to eliminate *kala-azar* from the Indian subcontinent, India, Bangladesh, and Nepal signed a tripartite memorandum of understanding in 2005 to achieve the elimination of VL as a public health problem. The elimination target was defined as reducing the annual incidence of VL to fewer than one case per 10,000 population at the sub-district (block) level in India and Bangladesh and at the district level in Nepal [5]. Subsequently, the programme evolved into the National Kala-Azar Elimination Programme (NKAEP), with strengthened strategies focusing on early diagnosis, complete treatment, active surveillance, vector control, and programme monitoring. The National Health Policy 2002 initially set a target for *kala-azar* elimination by 2010; however, this timeline was later revised to 2015. Continued political commitment and implementation of successive national roadmaps, operational guidelines, and accelerated action plans have enabled sustained progress towards elimination, with the programme currently being coordinated through the National Centre for Vector Borne Diseases Control (NCVBDC) [6,7].

West Bengal is one of the historically endemic states for *kala-azar* in India, and Malda district has long been recognized as an important focus of transmission. Following the implementation of intensified surveillance, improved case management, active case detection, vector control measures, and strengthened programme monitoring under the National Roadmap for Kala-azar Elimination (NRKE), all endemic blocks of Malda district achieved the elimination target by 2016 [8]. However, sustaining elimination remains challenging because of the continued occurrence of sporadic VL cases, the emergence of PKDL cases, population mobility, and the risk of disease resurgence. Continuous surveillance and periodic programme evaluation are therefore necessary to monitor progress and identify gaps in implementation [9].

The present study was undertaken to assess epidemiological trends of VL and PKDL and to evaluate the performance of key components of the NKAEP, including surveillance, vector control, and treatment outcomes in Malda district, West Bengal, during the period 2018-2022.

## Materials and methods

Study design

This retrospective descriptive study involved a secondary analysis of routinely collected programme data from the NKAEP in Malda district, West Bengal, India.

Data source

The study utilized secondary data routinely collected under the NKAEP. Data were obtained from records maintained at the District Kala-Azar Cell, Office of the Chief Medical Officer of Health (CMOH), Malda. Data sources included district *kala-azar* line lists, active case search reports, indoor residual spraying (IRS) reports, treatment records, logistics and commodity management registers, and surveillance and monitoring reports. Data pertaining to the period from January 2018 to 15 November 2022 were analysed. For selected programme indicators, available historical data from earlier years were also reviewed to examine long-term trends where relevant.

Study population

The study included all VL and PKDL cases recorded in the district programme database during the study period, based on the operational case definitions of the NVBDCP (now National Centre for Vector Borne Diseases Control) and the World Health Organization (WHO). As this study analysed the complete district programme database rather than a sampled population, no sampling technique or sample size calculation was applicable.

Variables and programme performance indicators

The analysis included epidemiological variables such as the number of VL and PKDL cases, incidence, demographic characteristics, and temporal trends. Programme performance indicators included surveillance activities, active case search, IRS, treatment outcomes, logistics and commodity management, and other monitoring indicators routinely collected under the NKAEP. Definitions of cases and programme indicators followed the operational guidelines of the NVBDCP and WHO.

Data management and quality assurance

Data from multiple programme records were compiled into a master database. Before analysis, the dataset was reviewed for completeness, consistency, and duplicate entries through cross-verification across the available programme records. Any discrepancies identified during compilation were reconciled using the corresponding district records to ensure data quality. Only anonymized programme data were analysed.

Statistical analysis

Data cleaning and statistical analysis were performed using Microsoft Excel 2019 (Microsoft Corporation, Redmond, WA). Descriptive statistical methods were used to summarize the findings. Frequencies, proportions, percentages, incidence rates, and temporal trends were calculated and presented using tables, charts, and graphical representations to describe epidemiological patterns and programme performance over the study period. Inferential statistical analyses were not performed because the study represented a complete enumeration of routinely reported programme records from the district rather than a sampled population, and the primary objective was descriptive assessment of epidemiological trends and programme performance.

Ethical considerations

The study was conducted using anonymized secondary programme data routinely collected as part of public health surveillance under the NKAEP. No patient identifiers were accessed during data extraction or analysis, and confidentiality was maintained throughout the study. As the analysis involved routinely collected programme data used for programme evaluation without any direct participant involvement or intervention, institutional ethics committee approval was not required in accordance with applicable institutional and national guidelines.

## Results

Epidemiological trends of kala-azar

**Figure 1:**
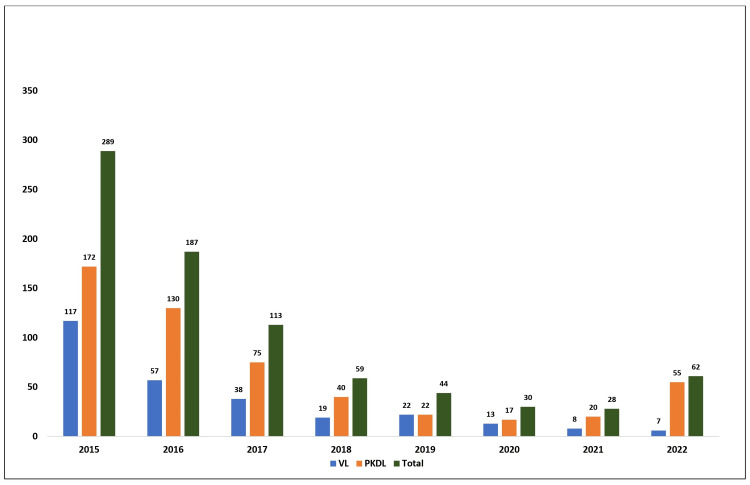
Trends of VL, PKDL, and total kala-azar cases in Malda district (2015-2022). VL: visceral leishmaniasis; PKDL: post-kala-azar dermal leishmaniasis.

A marked decline in the burden of *kala-azar* was observed in Malda district during the study period. The total number of reported *kala-azar* cases decreased from 289 in 2015 to 28 in 2021. However, an increase was observed in 2022, when 61 cases were reported up to 15 November. This increase was primarily attributable to a rise in PKDL cases, while VL cases continued to decline. The number of VL cases decreased steadily from 117 in 2015 to only six cases in 2022, indicating sustained progress towards elimination. In contrast, PKDL cases constituted an increasing proportion of the total disease burden during recent years, with the highest number reported in 2022 (Figure 1).

Demographic characteristics

Most *kala-azar* cases occurred among adults aged more than 15 years. During 2018-2022, 62 of 69 VL cases (89.9%) and 142 of 154 PKDL cases (92.2%) were reported among individuals aged >15 years. Only three VL cases were reported among children aged below five years, and no PKDL case was reported in this age group. Similarly, very few cases were reported among individuals aged 6-15 years (Table 1).

**Table 1:** Age-wise distribution of VL and PKDL cases in Malda district (2018-2022). Data are presented as n (%). Percentages represent the age distribution within each year (row percentages) for VL and PKDL separately. * Data for 2022 were available up to 15 November 2022. VL: visceral leishmaniasis; PKDL: post-kala-azar dermal leishmaniasis.

Year	Visceral leishmaniasis (VL)				Post-kala-azar dermal leishmaniasis (PKDL)			
	0-5 years, n(%)	6-15 years, n(%)	>15 years, n(%)	Total	0-5 years, n(%)	6-15 years, n(%)	>15 years, n(%)	Total
2018	1 (5.26)	2 (10.53)	16 (84.21)	19	0 (0.00)	2 (5.00)	38 (95.00)	40
2019	0 (0.00)	2 (9.09)	20 (90.91)	22	0 (0.00)	4 (18.18)	18 (81.82)	22
2020	1 (7.69)	0 (0.00)	12 (92.31)	13	0 (0.00)	1 (5.88)	16 (94.12)	17
2021	1 (12.50)	0 (0.00)	7 (87.50)	8	0 (0.00)	0 (0.00)	20 (100.00)	20
2022*	0 (0.00)	0 (0.00)	7 (100.00)	7	0 (0.00)	5 (9.09)	50 (90.91)	55
Total	3 (4.35)	4 (5.80)	62 (89.85)	69	0 (0.00)	12 (7.79)	142 (92.21)	154

Males accounted for a greater proportion of cases than females in most years. The proportion of male cases ranged from 46.4% to 64.4% during the study period. Female cases exceeded male cases only in 2021, when females accounted for 53.6% of all reported cases (Table 2).

**Table 2:** Demographic and clinical characteristics of reported leishmaniasis cases by year (2014-2022). Data are presented as n (%). Percentages for sex, tribal status, deaths, and VL were calculated using the total number of reported leishmaniasis cases (visceral leishmaniasis + post-kala-azar dermal leishmaniasis) in the corresponding year as the denominator. † Percentages for HIV–VL co-infection were calculated using the number of confirmed VL cases in the corresponding year as the denominator. * Data for 2022 were available up to 15 November 2022. VL: visceral leishmaniasis; PKDL: post-kala-azar dermal leishmaniasis; NA: data not available.

Year	Male, n (%)	Female, n (%)	Tribal cases, n (%)	Deaths, n (%)	Confirmed VL cases, n (%)	HIV–VL co-infection, n (%)^†^
2014	121 (53.3)	106 (46.7)	149 (65.64)	0 (0)	NA	NA
2015	169 (58.48)	120 (41.52)	202 (69.9)	0 (0)	117 (40.48)	1 (0.85)
2016	108 (57.14)	81 (42.86)	122 (64.55)	0 (0)	57 (30.16)	1 (1.75)
2017	69 (61.06)	44 (38.94)	62 (54.87)	0 (0)	38 (33.63)	1 (2.63)
2018	38 (64.41)	21 (35.59)	35 (59.32)	1 (1.69)	19 (32.2)	0 (0)
2019	24 (54.55)	20 (45.45)	25 (56.82)	0 (0)	19 (43.18)	0 (0)
2020	19 (63.33)	11 (36.67)	12 (40)	0 (0)	13 (43.33)	3 (23.08)
2021	13 (46.43)	15 (53.57)	20 (71.43)	0 (0)	8 (28.57)	0 (0)
2022*	37 (59.68)	25 (40.32)	36 (58.06)	2 (3.23)	7 (11.29)	2 (28.57)

Tribal populations contributed substantially to the disease burden. Between 40% and 71% of annual *kala-azar* cases were reported from tribal communities. The highest proportion of tribal cases was observed in 2021 (71.4%), while the lowest proportion was recorded in 2020 (40.0%) (Table 2). Habibpur, Gazole, Old Malda, and Bamongola, which have substantial tribal populations, consistently contributed a large share of cases.

Mortality and HIV-VL co-infection

*Kala-azar*-related mortality remained low throughout the study period. Only two deaths were reported between 2018 and 2022, one each in 2018 and 2022. No deaths were reported in 2019, 2020, or 2021 (Table 2).

All confirmed VL cases underwent HIV screening. HIV-VL co-infection was uncommon. Three HIV-VL co-infected cases were reported among 13 VL cases in 2020, while two co-infected cases were reported among seven VL cases in 2022. No HIV-VL co-infection was detected during 2018, 2019, or 2021 (Table 2).

Geographic distribution and elimination status

**Figure 2:**
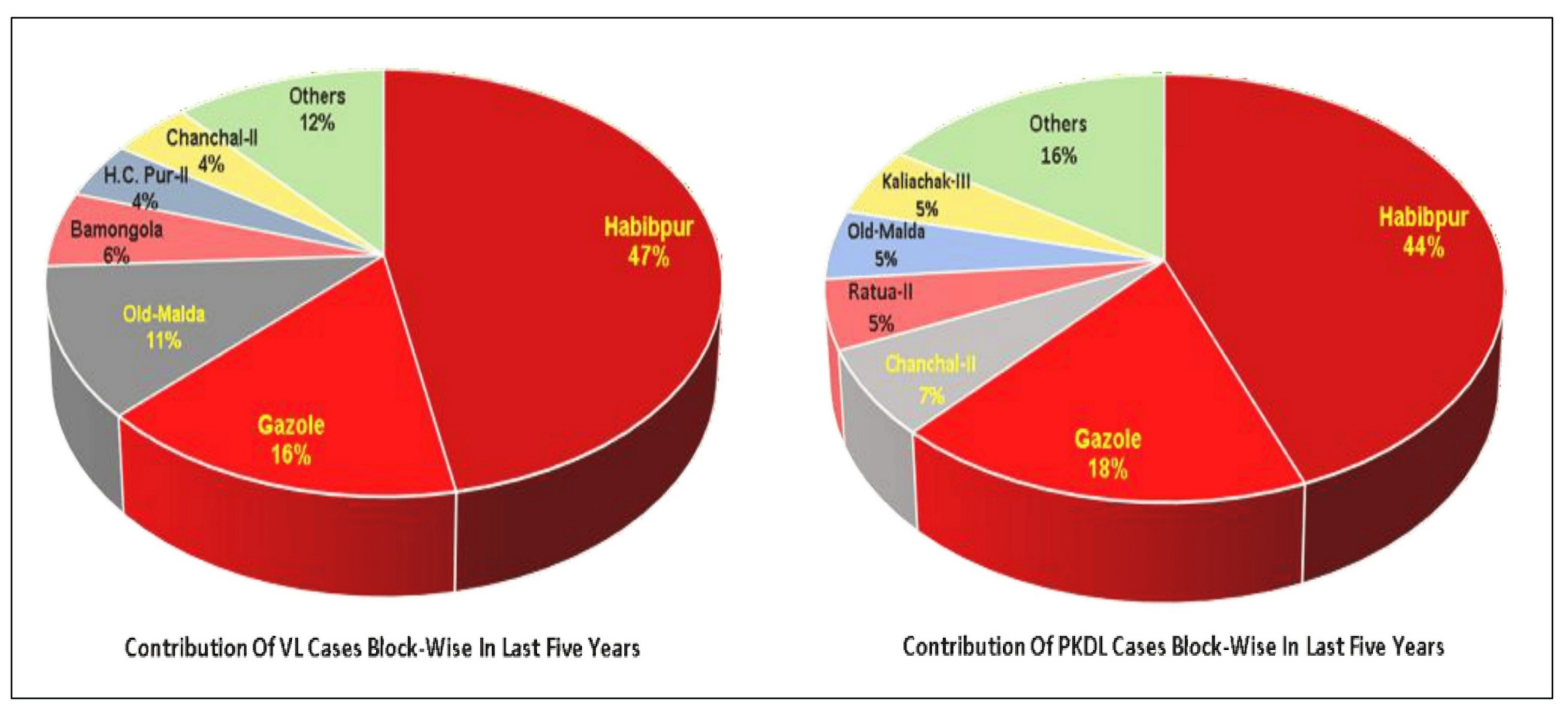
Block-wise contribution of VL and PKDL cases in Malda district (2018-2022). VL: visceral leishmaniasis; PKDL: post-kala-azar dermal leishmaniasis.

Considerable geographic heterogeneity in disease occurrence was observed across blocks. Habibpur contributed the highest proportion of both VL (47%) and PKDL (44%) cases during 2018-2022, followed by Gazole, which contributed 16% of VL cases and 18% of PKDL cases. Old Malda contributed 11% of VL cases, while Chanchal-II contributed 7% of PKDL cases (Figure 2).

Three blocks, Chanchal-I, Kaliachak-I, and Kaliachak-II, reported no VL or PKDL cases during the study period. Furthermore, Ratua-II and English Bazar did not report any VL cases between 2018 and 2022.

**Figure 3:**
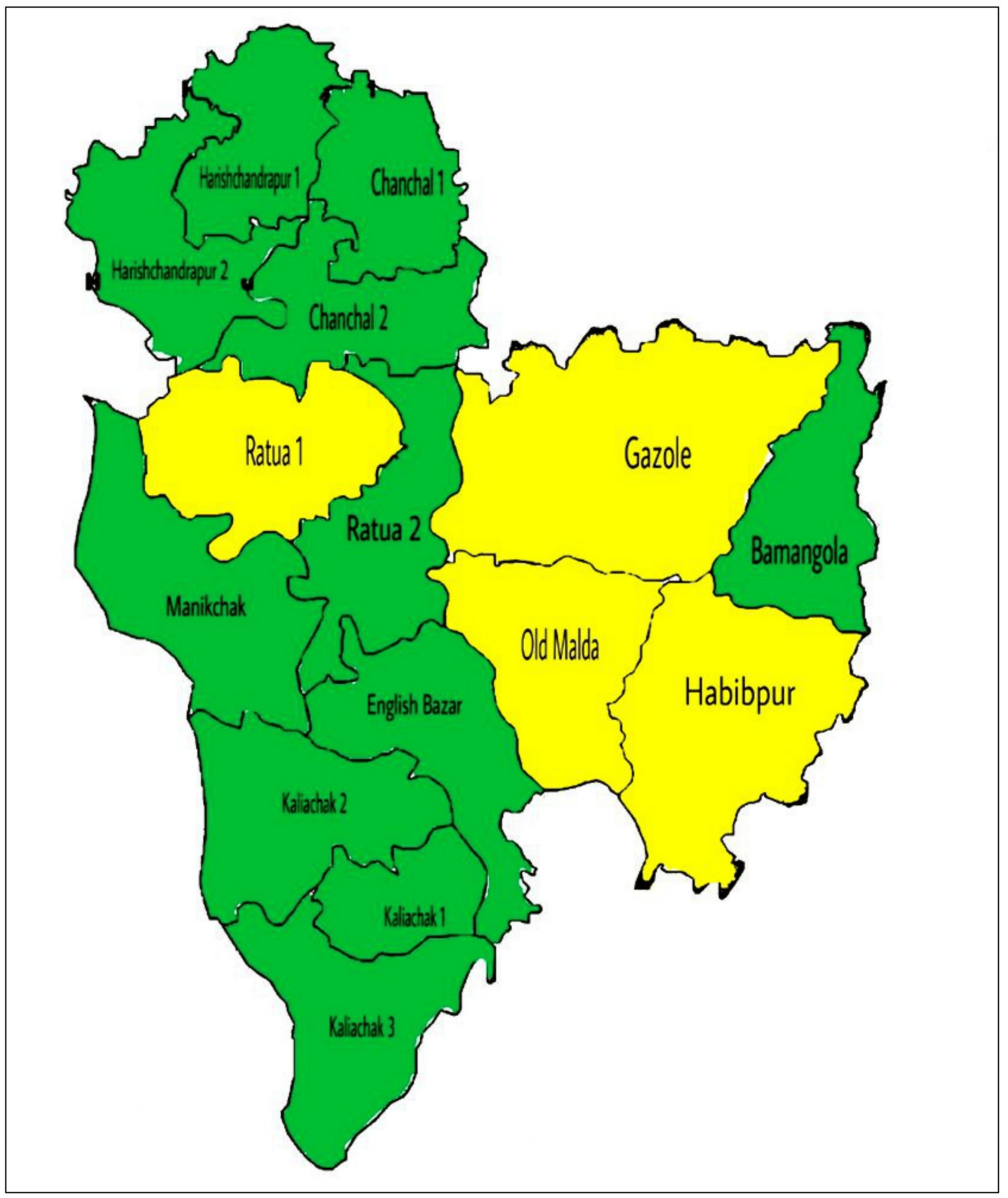
Block-wise incidence rate of VL per 10,000 population in Malda district (2022). Green: No cases. Yellow: Blocks reporting visceral leishmaniasis (VL) cases.

Despite continued case reporting from a few endemic blocks, all blocks maintained the elimination target of fewer than one VL case per 10,000 population. In 2022, 11 of the 15 blocks reported no VL cases. The remaining four blocks (Habibpur, Gazole, Old Malda, and Ratua-II) reported VL cases, but their incidence remained below the elimination threshold (Figure 3).

The number of affected villages and hotspot villages showed a declining trend over time. In 2022, a total of 62 *kala-azar* cases were reported from 59 villages, of which only three villages qualified as hotspot villages (≥2 cases per village). The number of persistent villages reporting cases also declined over the years, indicating contraction of transmission foci.

Treatment outcomes

Treatment outcomes were generally satisfactory throughout the study period. Treatment completion and final cure rates reached 100% in 2020. Although treatment completion remained high in 2021 (92.9%), the final cure rate declined to 65.4%.

Relapse cases demonstrated a declining trend from 2017 to 2020. However, a modest increase was observed subsequently, with seven relapse cases reported in 2021 and six cases reported in 2022.

Surveillance and active case detection

Active case search activities remained a key component of the elimination programme. Between 2017 and 2022, large populations were screened annually for symptoms suggestive of VL and PKDL. The proportion of confirmed cases among suspected cases ranged from 1.08% in 2020 to 2.82% in 2019 (Table 3).

**Table 3:** Active case search performance in Malda district (2017-2022). Data are presented as counts (n) unless otherwise indicated. Suspected case rate was calculated as the number of suspected cases per lakh (100,000) population screened. Confirmation rate (%) was calculated as (confirmed cases ÷ suspected cases) × 100. For 2019, only data from Harishchandrapur I and Harishchandrapur II (HC Pur I & II) blocks were available, reflecting localized surveillance and case detection activities. The limited geographical coverage should be considered when interpreting comparisons with other years. * Data for 2022 were available up to 15 November 2022.

Year	Total population screened	Total suspected cases	Total confirmed cases	Suspected cases per lakh population	% of confirmed cases out of suspected cases
2017	567486	1646	41	290.05	2.49
2018	282203	746	9	264.35	1.21
2019 (HC Pur I & II)	6369	142	4	2229.55	2.82
2020	306002	833	9	272.22	1.08
2021	438460	835	11	190.44	1.32
2022*	137091	288	5	210.08	1.74

The highest suspected case detection rate was observed in 2019 (2229.6 suspected cases per lakh population), although screening that year was limited to Harishchandrapur-I and Harishchandrapur-II blocks. In 2022, 137,091 individuals were screened, resulting in the identification of 288 suspected cases and five confirmed cases. These findings indicate continued surveillance efforts despite the declining disease burden.

Temporal analysis of case occurrence revealed seasonal variation in case detection. Peaks were generally observed during March-April and June-July. A pronounced increase in case detection during September 2022 coincided with intensified active case search activities.

Vector control and logistics management

IRS activities were conducted regularly in endemic areas throughout the study period. During the first round of IRS, population protection ranged from 91.3% to 93.6%, house coverage ranged from 89.3% to 94.5%, and room coverage ranged from 85.6% to 88.9%. During the second round, population protection and house coverage remained above 91% in all years except 2020, when the second round could not be conducted because of delays associated with the COVID-19 pandemic. Room coverage remained approximately 86-88% during the years in which the second round was implemented (Table 4).

**Table 4:** Indoor residual spray (IRS) performance report of Malda, 1st and 2nd round (2018-2022).

Year	1st round						2nd round					
	Population		Houses		Rooms		Population		Houses		Rooms	
	Targeted	% Protected	Targeted	% Covered	Targeted	% Covered	Targeted	% Population	Target	% Covered	Targeted	% Covered
2018	316210	93.13	63344	94.49	189831	88.09	324634	92.48	64920	93.13	184192	86.15
2019	228519	91.28	49249	92.31	135103	88.91	234625	93.43	48365	92.46	130120	87.95
2020	176713	93.62	36966	93.65	111159	86.66	Not done	Not done	Not done	Not done	Not done	Not done
2021	189824	93.57	39813	93.41	119993	88.67	191335	93.4	40226	93.92	123477	87.92
2022	70759	93.13	15447	89.25	45303	85.63	96111	91.29	20045	91.27	61705	86.94

The district maintained uninterrupted availability of essential *kala-azar* programme commodities throughout the study period. Adequate stocks of liposomal amphotericin B (AmBisome), miltefosine, rK39 rapid diagnostic kits, and synthetic pyrethroid insecticides were available at all times. Although the entire stock of synthetic pyrethroid insecticide was distributed in 2021, no stock-outs of diagnostic, therapeutic, or vector-control commodities occurred.

## Discussion

The present study demonstrates sustained achievement of *kala-azar* elimination targets in Malda district during 2018-2022. The progressive decline in VL cases and maintenance of incidence below one case per 10,000 population at the block level reflect the effectiveness of the NKAEP, which emphasizes early diagnosis, prompt treatment, active surveillance, and vector control interventions. These findings are consistent with the broader progress reported from the Indian subcontinent, where sustained programmatic efforts have led to substantial reductions in VL incidence and have enabled many endemic districts to achieve elimination targets [9-11].

Although VL cases continued to decline, an increase in PKDL cases was observed in 2022. This finding is programmatically important because PKDL patients are recognized as an important human reservoir for *Leishmania donovani* transmission. Previous studies have demonstrated the infectious potential of PKDL patients to sandflies and highlighted their role in sustaining transmission in low-endemic settings [3,4]. Enhanced detection of PKDL cases may, in part, reflect intensified surveillance activities, active case-finding initiatives, increased awareness among healthcare providers and communities, and improved diagnostic efforts implemented during the consolidation phase of the elimination programme. In addition, PKDL can develop months to years after successful treatment of VL; therefore, the increase observed in 2022 may represent delayed manifestation among previously treated VL patients rather than renewed transmission. Similar observations have been reported from other endemic regions, where declining VL incidence has been accompanied by increased identification of PKDL cases following strengthened surveillance and active case detection [12,13]. However, because this study was based on routinely collected secondary programme data, it was not possible to determine the relative contribution of these factors or establish causal explanations for the observed increase in PKDL. Further longitudinal and analytical studies incorporating individual-level clinical and epidemiological data are warranted to better understand the determinants of PKDL occurrence and to inform strategies for sustaining *kala-azar* elimination.

The predominance of *kala-azar* among adults aged above 15 years in the present study is comparable with findings from previous epidemiological investigations conducted in endemic areas of India and neighbouring countries. Adults are more likely to experience occupational and environmental exposure to sandfly habitats, thereby increasing their risk of infection [14,15]. The very low number of cases among young children may indicate improved household awareness, healthcare access, and surveillance coverage.

A substantial proportion of cases occurred among tribal populations, particularly in blocks such as Habibpur, Gazole, and Bamongola. This pattern is consistent with existing evidence linking *kala-azar* transmission to socioeconomic deprivation, poor housing conditions, malnutrition, and limited access to healthcare services [15-17]. Previous studies have reported clustering of *kala-azar* in marginalized and underserved communities where environmental conditions favour sandfly breeding and human-vector contact. The persistence of cases in tribal-dominated areas suggests that targeted social and environmental interventions may be necessary to sustain elimination gains.

Mortality due to *kala-azar* remained extremely low throughout the study period, with only two reported deaths. This finding likely reflects improvements in case detection and treatment availability following the adoption of highly effective regimens, including single-dose liposomal amphotericin B, which has substantially improved treatment outcomes in endemic regions [18]. High treatment completion rates observed in the district further support the effectiveness of current case-management practices.

Although HIV-VL co-infection was uncommon, its occurrence remains epidemiologically important because co-infected individuals are at increased risk of relapse, treatment failure, and mortality. Previous reviews have highlighted HIV co-infection as a significant challenge for *kala-azar* control and elimination efforts [2]. Continued integration of *kala-azar* and HIV services, including routine HIV screening of VL patients and long-term follow-up, remains essential.

The present analysis highlights the importance of active case search in sustaining elimination. Community-based screening activities continued to identify suspected and confirmed cases despite low overall incidence. Similar findings have been reported from elimination settings where active surveillance has been critical for detecting residual transmission foci and preventing resurgence [10,19]. Seasonal peaks in case detection observed during periods of intensified active case search further emphasize the value of targeted surveillance strategies.

IRS coverage remained consistently high during the study period, indicating strong operational performance and community participation. Vector control has been recognized as a cornerstone of *kala-azar* elimination strategies in South Asia, and sustained IRS coverage has contributed substantially to reducing transmission [5,6,20]. Although room coverage remained slightly lower than household coverage, likely due to migration-related absenteeism, overall coverage levels were consistent with programmatic recommendations.

The successful maintenance of elimination status in all blocks of Malda district demonstrates the effectiveness of an integrated strategy combining surveillance, case management, vector control, and logistics support. Nevertheless, the persistence of PKDL cases, occurrence of HIV-VL co-infection, and concentration of disease among tribal populations highlight the need for continued vigilance. Sustained surveillance, targeted interventions in high-risk communities, and strengthened monitoring systems will be essential to prevent recrudescence and achieve long-term interruption of transmission [7,21].

Strengths and limitations

This study has several notable strengths. It utilized comprehensive district-level routine surveillance and programme data collected under the NKAEP, enabling a comprehensive assessment of epidemiological trends and programme performance across all endemic blocks of Malda district. By incorporating multiple programme components, including disease surveillance, active case detection, treatment outcomes, IRS, and logistics and commodity management, the study provides a comprehensive overview of programme implementation under real-world public health conditions. In addition, the analysis spans a five-year period following the achievement of the *kala-azar* elimination target, allowing evaluation of the district's progress in sustaining elimination while identifying persistent challenges, such as PKDL, relapse cases, and the need for continued surveillance.

Several limitations should also be considered when interpreting the findings. First, the study was based on routinely collected secondary programme data, which may be subject to under-reporting, reporting delays, incomplete records, data-entry errors, and variations in data quality across reporting units. Second, the use of aggregated programme data precluded individual-level analyses, limiting the ability to evaluate patient-specific risk factors, temporal progression from VL to PKDL, or associations between clinical and demographic characteristics, including HIV co-infection. Consequently, inferential statistical analyses to identify predictors or causal relationships were beyond the scope of the present study. Third, although the findings provide valuable evidence for programme monitoring and evaluation, they are derived from a single endemic district and may not be directly generalizable to other endemic regions with different epidemiological or health system contexts. Finally, some programme indicators were dependent on the completeness and accuracy of routine reporting and documentation, which may have influenced the precision of the estimated programme performance measures. The routine programme database did not permit longitudinal linkage of individual VL patients with subsequent PKDL occurrence; therefore, this proportion could not be estimated.

Despite these limitations, the study provides programmatic evidence on the sustainability of *kala-azar* elimination at the district level and demonstrates the value of routinely collected surveillance data for monitoring epidemiological trends, evaluating programme performance, and informing public health planning and policy decisions in the post-elimination phase.

## Conclusions

The Malda district of West Bengal, India, has successfully sustained the *kala-azar* elimination target across all endemic blocks since 2016, demonstrating the effectiveness of the NKAEP in reducing and maintaining low levels of VL transmission. The continued decline in VL cases, low mortality, high treatment completion rates, active case detection, and consistent vector control activities reflect strong programme implementation and surveillance systems. However, the increasing detection of PKDL cases highlights the need for continued vigilance, as PKDL patients may serve as reservoirs for ongoing transmission. Sustaining elimination will require strengthened surveillance and follow-up of VL patients, early detection and treatment of PKDL, maintenance of high-quality vector control measures, and targeted interventions in tribal and historically endemic areas. Continued monitoring and programmatic commitment are essential to prevent disease resurgence and achieve long-term interruption of *kala-azar* transmission.

## Appendices

**Table 5:** Operational definitions and calculation of epidemiological and programme performance indicators used in the study. VL: visceral leishmaniasis; PKDL: post-kala-azar dermal leishmaniasis; IRS: indoor residual spraying; NKAEP: National Kala-Azar Elimination Programme. * Population denominators were obtained from official district population estimates used by the National Kala-Azar Elimination Programme for routine surveillance and programme monitoring.

Indicator	Operational definition	Calculation/measurement	Data source
Visceral leishmaniasis (VL) case	A confirmed VL case as defined by the National Kala-Azar Elimination Programme (NKAEP).	Number of confirmed VL cases reported during the specified period.	District kala-azar line list, treatment records
Post-kala-azar dermal leishmaniasis (PKDL) case	A confirmed PKDL case according to NKAEP.	Number of confirmed PKDL cases reported during the specified period.	District kala-azar line list, treatment records
Annual incidence of VL	Number of new VL cases occurring in the population during a calendar year.	(Number of new VL cases ÷ Mid-year population) × 10,000 population*	District surveillance records; population estimates
Block attaining the elimination target	Administrative block reporting an annual VL incidence below the programme elimination threshold.	Annual VL incidence <1 case per 10,000 population at the block level.	District surveillance database
Active case search (ACS)	House-to-house search conducted in endemic villages to identify individuals with prolonged fever suggestive of VL.	Number of villages/households covered and suspected cases identified according to programme records.	Active case search reports
Indoor residual spraying (IRS) coverage	Proportion of targeted households sprayed during an IRS round.	(Number of households sprayed ÷ Number of households targeted) × 100	IRS implementation reports
IRS rounds completed	Number of planned IRS rounds conducted during the reporting year.	Reported as frequency and completion status.	IRS reports
Treatment initiation	Confirmed VL/PKDL cases started on recommended treatment.	(Number initiated on treatment ÷ Total confirmed cases) × 100	Treatment registers
Treatment completion	Patients completing the prescribed treatment regimen.	(Number completing treatment ÷ Number initiated on treatment) × 100	Treatment registers
Initial cure	Patients fulfilling programme criteria for cure at completion of treatment.	(Number achieving initial cure ÷ Number completing treatment) × 100	Treatment outcome records
Relapse	Previously treated VL patient developing recurrent disease according to programme criteria.	Number and proportion of relapse cases among treated patients.	Follow-up records
Mortality (case fatality)	Death among confirmed VL patients during treatment or programme-defined follow-up.	(Number of deaths ÷ Total confirmed VL cases) × 100	Treatment and surveillance records
Logistics and commodity availability	Availability of essential diagnostics, drugs, insecticides, and programme supplies at the district level.	Assessed descriptively from stock and logistics records.	Commodity management registers
Surveillance performance	Timeliness and completeness of routine case reporting and monitoring activities.	Assessed descriptively using district surveillance reports.	Surveillance and monitoring reports
Epidemiological trend	Change in disease occurrence over the study period.	Annual frequencies, proportions, incidence rates, and graphical trend analysis.	Compiled programme database

## References

[1] Ghosh C, Karmakar C, Sarkar D (2026). Post-kala-azar dermal leishmaniasis: a global overview. Clin Exp Dermatol.

[2] Gill N, Pandey D, Roy N, Dhingra N, Jain S (2021). Kala-azar in India - progress and challenges towards its elimination as a public health problem. Wkly Epidemiol Rec.

[3] Zijlstra EE, Musa AM, Khalil EA, El-Hassan IM, El-Hassan AM (2003). Post-kala-azar dermal leishmaniasis. Lancet Infect Dis.

[4] Mondal D, Bern C, Ghosh D (2019). Quantifying the infectiousness of post-kala-azar dermal leishmaniasis toward sand flies. Clin Infect Dis.

[5] WHO Regional Office for South-East Asia (2016). Regional Strategic Framework for Elimination of Kala-Azar From the South-East Asia Region (2011-2015).

[6] Directorate of National Vector Borne Disease Control Programme (2022). World Health Organization. Leishmaniasis. Accelerated Plan for Kala-azar Elimination 2017.

[7] National Vector Borne Disease Control Programme (2015). National Centre for Vector Borne Diseases Control. National Kala-Azar Elimination Programme: Operational Guidelines [Internet]. New Delhi: Directorate General of Health Services, Ministry of Health and Family Welfare, Government of India. Operational Guidelines on Kala-Azar (Visceral Leishmaniasis) Elimination in India - 2015.

[8] National Centre for Vector Borne Diseases Control Programme (2018). Press Information Bureau, Government of India, Ministry of Health and Family Welfare. Elimination of kala azar. National Kala-azar Elimination Programme Operational Guidelines.

[9] Hirve S, Kroeger A, Matlashewski G (2017). Towards elimination of visceral leishmaniasis in the Indian subcontinent—translating research to practice to public health. PLoS Negl Trop Dis.

[10] Control of Neglected Tropical Diseases (NTD) (2020). World Health Organization. Ending the neglect to attain the Sustainable Development Goals: road map. Ending the Neglect to Attain the Sustainable Development Goals: A Road Map for Neglected Tropical Diseases 2021-2030.

[11] World Health Organization, Regional Office for South-East Asia (2020). National Center for Vector Borne Diseases Control. Kala-azar situation in India: annual report. Independent Assessment of Kala-Azar Elimination Programme India.

[12] World Health Organization (2013). World Health Organization. Post-kala-azar dermal leishmaniasis: a manual for case detection and management. Post-Kala-Azar Dermal Leishmaniasis: A Manual for Case Management and Control.

[13] Rishav Rishav, Ahmed N, Raj Y, Deepika PSVM, Lahkar M (2026). Post-kala-azar dermal leishmaniasis: a systematic review of immunopathogenesis, molecular diagnostics, and public health impact. J Parasit Dis.

[14] Singh SP, Reddy DC, Mishra RN, Sundar S (2006). Knowledge, attitude, and practices related to Kala-azar in a rural area of Bihar state, India. Am J Trop Med Hyg.

[15] Bern C, Courtenay O, Alvar J (2010). Of cattle, sand flies and men: a systematic review of risk factor analyses for South Asian visceral leishmaniasis and implications for elimination. PLoS Negl Trop Dis.

[16] Alvar J, Vélez ID, Bern C (2012). Leishmaniasis worldwide and global estimates of its incidence. PLoS One.

[17] Boelaert M, Meheus F, Sanchez A (2009). The poorest of the poor: a poverty appraisal of households affected by visceral leishmaniasis in Bihar, India. Trop Med Int Health.

[18] Sundar S, Chakravarty J (2010). Liposomal amphotericin B and leishmaniasis: dose and response. J Glob Infect Dis.

[19] Banjara MR, Hirve S, Siddiqui NA (2012). Visceral leishmaniasis clinical management in endemic districts of India, Nepal, and Bangladesh. J Trop Med.

[20] Picado A, Das ML, Kumar V (2010). Effect of village-wide use of long-lasting insecticidal nets on visceral Leishmaniasis vectors in India and Nepal: a cluster randomized trial. PLoS Negl Trop Dis.

[21] World Health Organization South-East Asia Region (2010). World Health Organization. Framework for monitoring and evaluation of visceral leishmaniasis elimination in the South-East Asia Region. Indicators for Monitoring and Evaluation of the Kala-Azar Elimination Programme.

